# Technological advancements in cancer diagnostics: Improvements and limitations

**DOI:** 10.1002/cnr2.1764

**Published:** 2023-01-06

**Authors:** Akhil Pulumati, Anika Pulumati, Bilikere S. Dwarakanath, Amit Verma, Rao V. L. Papineni

**Affiliations:** ^1^ University of Missouri‐Kansas City Kansas City Missouri USA; ^2^ Central Research Facility Sri Ramachandra Institute of Higher Education and Research Porur Chennai India; ^3^ Department of Biotechnology Indian Academy Degree College Bangalore India; ^4^ PACT & Health LLC Branford Connecticut USA; ^5^ Department of Surgery University of Kansas Medical Center Kansas City Kansas USA

**Keywords:** 2‐fluoro‐2‐deoxy‐D‐glucose, artificial intelligence, computed tomography, magnetic resonance imaging, positron emission tomography

## Abstract

**Background:**

Cancer is characterized by the rampant proliferation, growth, and infiltration of malignantly transformed cancer cells past their normal boundaries into adjacent tissues. It is the leading cause of death worldwide, responsible for approximately 19.3 million new diagnoses and 10 million deaths globally in 2020. In the United States alone, the estimated number of new diagnoses and deaths is 1.9 million and 609 360, respectively. Implementation of currently existing cancer diagnostic techniques such as positron emission tomography (PET), X‐ray computed tomography (CT), and magnetic resonance spectroscopy (MRS), and molecular diagnostic techniques, have enabled early detection rates and are instrumental not only for the therapeutic management of cancer patients, but also for early detection of the cancer itself. The effectiveness of these cancer screening programs are heavily dependent on the rate of accurate precursor lesion identification; an increased rate of identification allows for earlier onset treatment, thus decreasing the incidence of invasive cancer in the long‐term, and improving the overall prognosis. Although these diagnostic techniques are advantageous due to lack of invasiveness and easier accessibility within the clinical setting, several limitations such as optimal target definition, high signal to background ratio and associated artifacts hinder the accurate diagnosis of specific types of deep‐seated tumors, besides associated high cost. In this review we discuss various imaging, molecular, and low‐cost diagnostic tools and related technological advancements, to provide a better understanding of cancer diagnostics, unraveling new opportunities for effective management of cancer, particularly in low‐ and middle‐income countries (LMICs).

**Recent Findings:**

Herein we discuss various technological advancements that are being utilized to construct an assortment of new diagnostic techniques that incorporate hardware, image reconstruction software, imaging devices, biomarkers, and even artificial intelligence algorithms, thereby providing a reliable diagnosis and analysis of the tumor. Also, we provide a brief account of alternative low cost‐effective cancer therapy devices (CryoPop®, LumaGEM®, MarginProbe®) and picture archiving and communication systems (PACS), emphasizing the need for multi‐disciplinary collaboration among radiologists, pathologists, and other involved specialties for improving cancer diagnostics.

**Conclusion:**

Revolutionary technological advancements in cancer imaging and molecular biology techniques are indispensable for the accurate diagnosis and prognosis of cancer.

## 1 INTRODUCTION

Increase in the rate of cancer incidence world‐wide combined with enhanced mortality in some of the malignancies continues to pose a challenge to biomedical scientific community for an effective management of cancer. Prevention being a realistic probability only in few types of cancers, technological advancements in cancer diagnostics with precise determination of location, size, stage, and molecular characteristics, is urgently needed for cancer treatment, due to a worldwide increase in cancer related mortality.^1^ Currently, the approach for diagnosis as a part of the clinical management of cancer includes a physical examination for abnormalities in various anatomical locations and a battery of laboratory investigations using blood and urine combined with a combination of radiologic and nuclear medicine based noninvasive imaging modalities like computerized X‐ray scan (popularly referred to as CT scan), ultrasonography (US), magnetic resonance imaging (MRI), bone scan, positron emission tomography (using FDG, PSMA etc) followed by minimally invasive biopsy (needle aspirations) or invasive (surgical) biopsy coupled with histo‐pathological examination to establish the identity and stage of the cancer. A number of immunological probes coupled with flow cytometric analysis are also widely used in the diagnosis and prognosis of liquid cancers namely, leukemias. While these approaches have been the backbone of diagnosis and treatment of cancers, they are nonspecific and also effective in moderately or highly advanced malignancies. Since early diagnosis of cancer has been found to improve the prognosis due to effectiveness of various therapies at this stage, and use of molecular targeted therapies significantly reduce the off‐target effects (or side effects), there is a great deal of effort in developing diagnostic probes or biomarkers and approaches that target specific molecular and genetic abnormalities as well as highly sensitive analytical capabilities.

**FIGURE 1:**
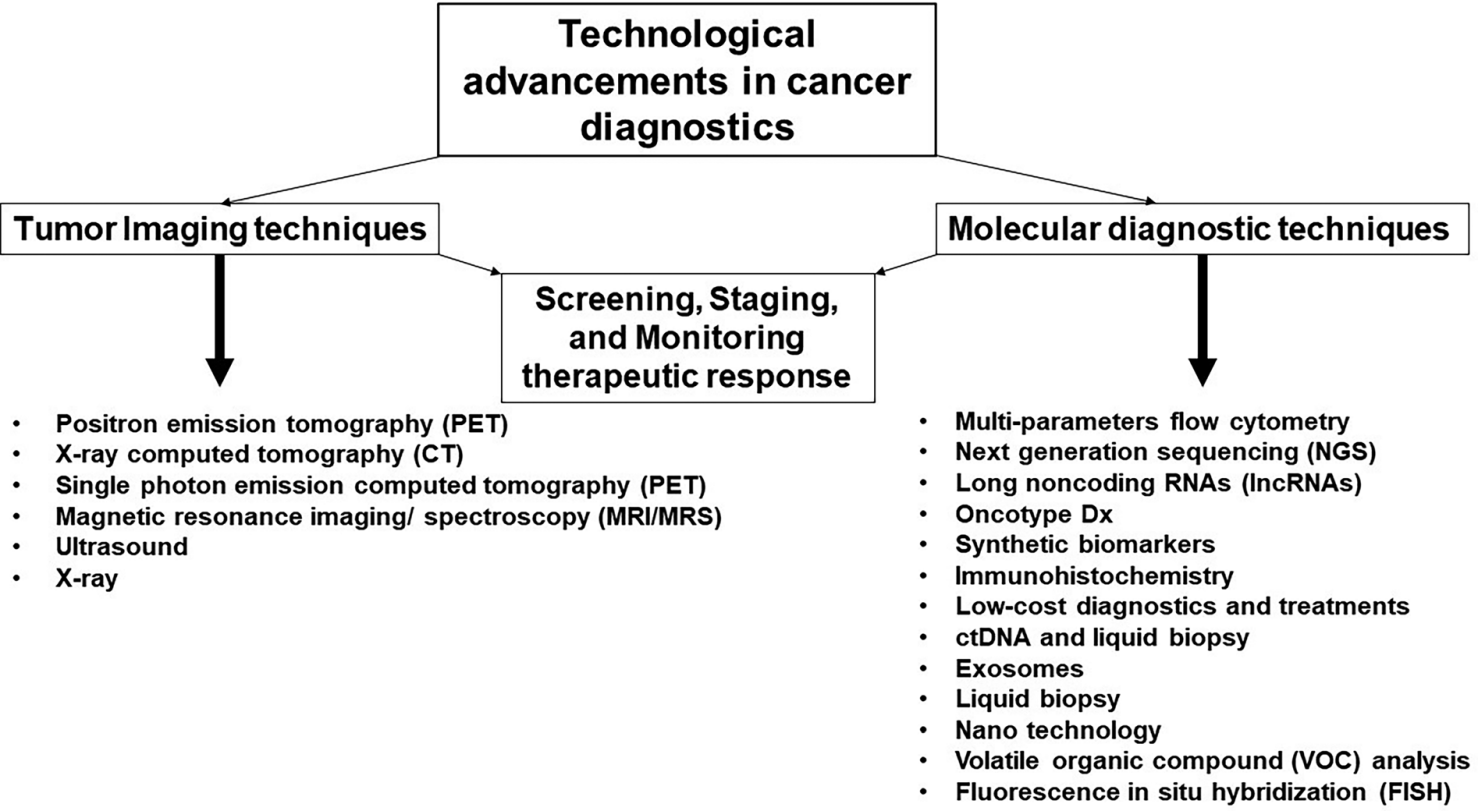
Technological advancements in cancer diagnostics

Cancer diagnosis is rapidly evolving due to continuous advancements in our knowledge of the disease and improvements in technology that increase the feasibility of reliable diagnostic approaches.^2^, ^3^ There are several cancer diagnostic modalities such as 2D and 3D imaging of tumors using positron emission tomography (PET), MRI, single photon emission computed tomography (SPECT), computed tomography (CT), X‐ray imaging, and analysis of molecular (metabolic, proteomic, genomic, and transcriptomic) signatures of cancer cells, thereby leveraging the cancer diagnosis and management (Figure 1).^4^ However there is a lack of clinical effectiveness and cost effectiveness of various cancer diagnostic modalities, besides having strategies/methods for evaluating associated risk and monitoring the therapeutic response.^5^ Imaging is the most widely used tool to identify a diverse category of cancers based on various phenotypic properties associated with tissues within the tumor.^6^ It is commonly used for the purposes of screening, staging, and monitoring tumor progression due to its accessibility and lack of invasiveness.^6^ It is important to understand that the effectiveness of an imaging modality is heavily dependent on the growth rate of the solid tumor which can be represented by several different mathematical models: exponential, logistic, linear, surface, Mendelsohn, Gompertz, and Bertalanffy model.^7^ The curve represented by the Gompertz model, for example, is a sigmoidal curve capturing the idea that a tumor's growth rate decreases as the mass of the tumor increases as a function of time. The reasoning behind this idea is that the proliferation of cancerous cells is highly dependent on the availability of factors such as nutrients and physical space, so as a tumor expands in size, the accessibility of these resources declines which ultimately leads to a slowed growth rate. Despite the existence of many tumor growth kinetic models, the Gompertz model has been shown to best represent solid tumors, primarily because it highlights a major characteristic of the vast majority of human cancers: they do not grow exponentially due to the doubling time, the number of days required for a tumor to double in volume, steadily increasing as the tumor grows rather than remaining constant. Modalities such as plain film X‐ray, CT, US, MRI, and PET are the most commonly used to provide information about the physical structure, metabolic activity, and functional status of the cancer in the clinical scenario Table 1.^6^ However, among each of these imaging modalities are inherent variations in resolution, sensitivity, and contrast generation which help to fulfill the primary principles and goal of cancer imaging: detection, characterization, and monitoring of tumors.^6^, ^8^ Detection refers to the localization of particular areas of interest within the image which allows for ability to characterize the tumor. Characterization refers to the triad of determining the diagnosis, stage, and prognosis of the tumor. Finally, monitoring refers to the process of observing how the tumor progresses and impacts the rest of the body over time. The implementation and enhancement of these three main principles of cancer detection is what has allowed for a decrease in diagnostic ambiguity and inaccuracy which has led to improved patient care and outcomes as a whole. Moreover, methods of enhancing the process of generating an adequate signal to background ratio and threshold for detection still remains an area of investigation, besides reducing the artifacts associated with physical and physiological motions such as anatomical barriers and imaging time.^6^, ^9^ Additionally, the imaging methods like plain X‐ray, CT, and PET uses ionizing radiation and radioactive material which warrants the need for advanced biodosimetry methods to prevent high dose radiation exposure during imaging.^10^, ^11^ By encompassing molecular diagnostic techniques, multi‐parameter flow cytometry, immunohistochemistry, microarray, next generation sequencing, and other related molecular biology techniques, and nanomedicine, the breadth of diagnostics has grown significantly over the years.^6^, ^12^, ^13^, ^14^, ^15^ Despite the benefits of molecular diagnostic techniques in tumor classification, characterization, and precision medicine have been clinically demonstrated, inter‐individual variations in the molecular signatures/pathways, validation methods, quality assurance, and high assay costs are some of the major limitations associated.^16^, ^17^, ^18^, ^19^, ^20^ In this article, we will discuss various imaging and molecular diagnostic techniques commonly used for the detection of cancer, as well as rationales behind their actions, efficacies, and advancements.

**TABLE 1:** Technologies used for diagnosis of various types of Cancer and associated advantages and limitations

Diagnostic technique	Measurement	Type of cancer detected	Advantages	Limitations	References
Positron Emission Tomography (PET)	Measures blood flow to various areas of a specific organ, allowing the construction of an image displaying which regions of the organ are more active at a particular time.	Brain, breast, cervical, colorectal, esophageal, head and neck, pulmonary, lymphatic, pancreatic, prostatic, skin, and thyroid tumors.	Can be performed in addition to a CT scan to provide both functional and anatomical information. May have diagnostic value in indentifying cancerous lesions that may have been missed on conventional imaging. Analyzes metastasis via lymph nodes more accurately than conventional imaging.	Limited spatial resolution and cancerous lesion Detectability. Radiation exposure via intravenous administration of radioactive compounds.	4, 6, 9, 10, 11, 21, 22, 23, 24, 25, 26, 27, 28, 29, 30, 31, 32, 33, 34, 35, 36, 37, 38, 39, 40, 41, 42, 43, 44, 45, 108
Computed Tomography (CT)	A series of X‐ray images taken from different angles around the body to construct cross‐sectional images of bones, vessels, and soft tissues.	Colorectal, gastric, head and neck, kidney, bone, bladder, ovarian tumors.	Fast scan with the potential to decrease motion artifacts. Cortical bone information used to create digitally reconstructed radiographs. Accurate spatial information.	Sub‐optimal soft tissue imaging. Radiation exposure. Lack of functional information.	4, 6, 21, 46, 47, 48, 49, 50, 51
Magnetic Resonance Imaging (MRI)	Use a magnetic field and a radio waves to construct detailed images of organs and tissues.	Brain, primary bone, soft tissue sarcomas, spinal cord, prostatic, bladder, uterine, and ovarian tumors.	Detailed soft tissue imaging. Lack of ionizing radiation exposure. Gadolinium, the contrast agent used in MRIs, is less likely to cause an allergic reaction compared to iodine‐based contrast agents used in X‐rays and CT.	Contraindicated in the presence of internal and external metal objects due to interference with the magnetic fields. Expensive. Time consuming. Must remain in an enclosed machine which can be problematic in claustrophobic patients.	6, 26, 52, 55, 61
Magnetic Resonance Spectroscopy (MRS)	Use a stronger magnetic field than MRIs to construct images depicting metabolism and blood flow.	Brain, breast, colorectal, prostatic, pancreatic, hepatobiliary, and gastric tumors.	Detailed soft tissue imaging. Lack of ionizing radiation exposure. Can obtain biological, anatomical, physiological, and metabolic information.	Time consuming. Expensive. Lack of anatomical information.	24, 26, 49, 52, 53, 54, 55, 56, 57, 58, 59, 60, 61

## 2 IMAGING TECHNIQUES

### 2.1 Positron emission tomography

Tomographic images produced by X‐ray absorbance, magnetic resonance properties, and ultrasound reflection are imaging techniques that function based on the structural properties of the tumor.^6^ Perhaps the most widely used imaging method present for the diagnosis of cancers is the PET, which is based on the functional status of the tumor tissue. A PET scan creates an image similar to a camera, but rather than creating an image by using visible light, it captures the simultaneous gamma rays generated by the annihilation of two positrons from a pharmaceutical agent that is localized differentially in the tumor tissue linked to its functional status to create an image.^21^, ^22^ First, certain isotopes of oxygen, nitrogen, and carbon generated in a cyclotron (a device that accelerates the particles) replace a hydrogen atom in a molecule of interest that emits a positron. A collision of a positron and electron occurs within the tissue, releasing gamma rays which are then detected by the PET scan.^21^ The complex mechanism utilized by this diagnostic tool provides the foundation, not only for the three principles of cancer detection, but also for the clinical decision‐making process regarding management according to a prospective cohort study executed by ​​the National Oncologic PET Registry (NOPR).^23^ This study ​​collected data on the cancer management plan prior to and following analyzing the findings indicated on the PET scan via questionnaires. The findings of this study concluded that the post‐PET management plan changed to monitoring and observing in 37% and treatment in 48% of the patients. In addition, about 70% of patients who were initially planning to undergo a biopsy were advised against that initial advice following completion of the PET scan. Finally, in patients whose management plan consisted of treatment prior to and following the PET scan, the post‐PET management strategy involved a significant change concerning the treatment type in 8.7% and treatment goals in 5.6%. Overall, physicians altered their initial management plan in 36.5% of cases due to the findings presented in the PET scan which exemplifies the great benefit provided by this imaging technique in oncologic settings.

**FIGURE 2:**
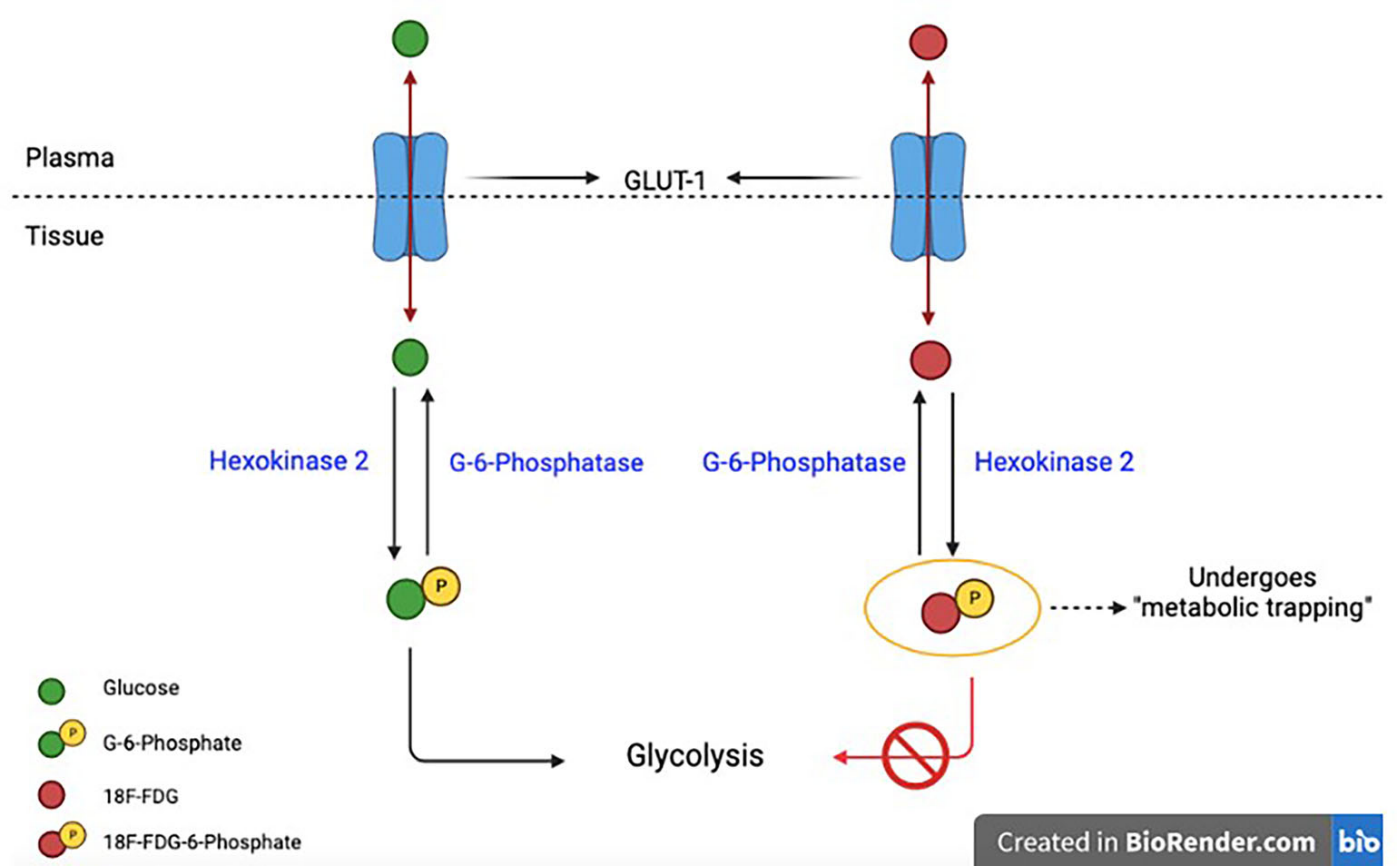
Molecular basis of 18fluorine‐2‐Deoxy‐D‐Glucose (FDG) in positron emission tomography (PET) diagnostic imaging

**FIGURE 3:**
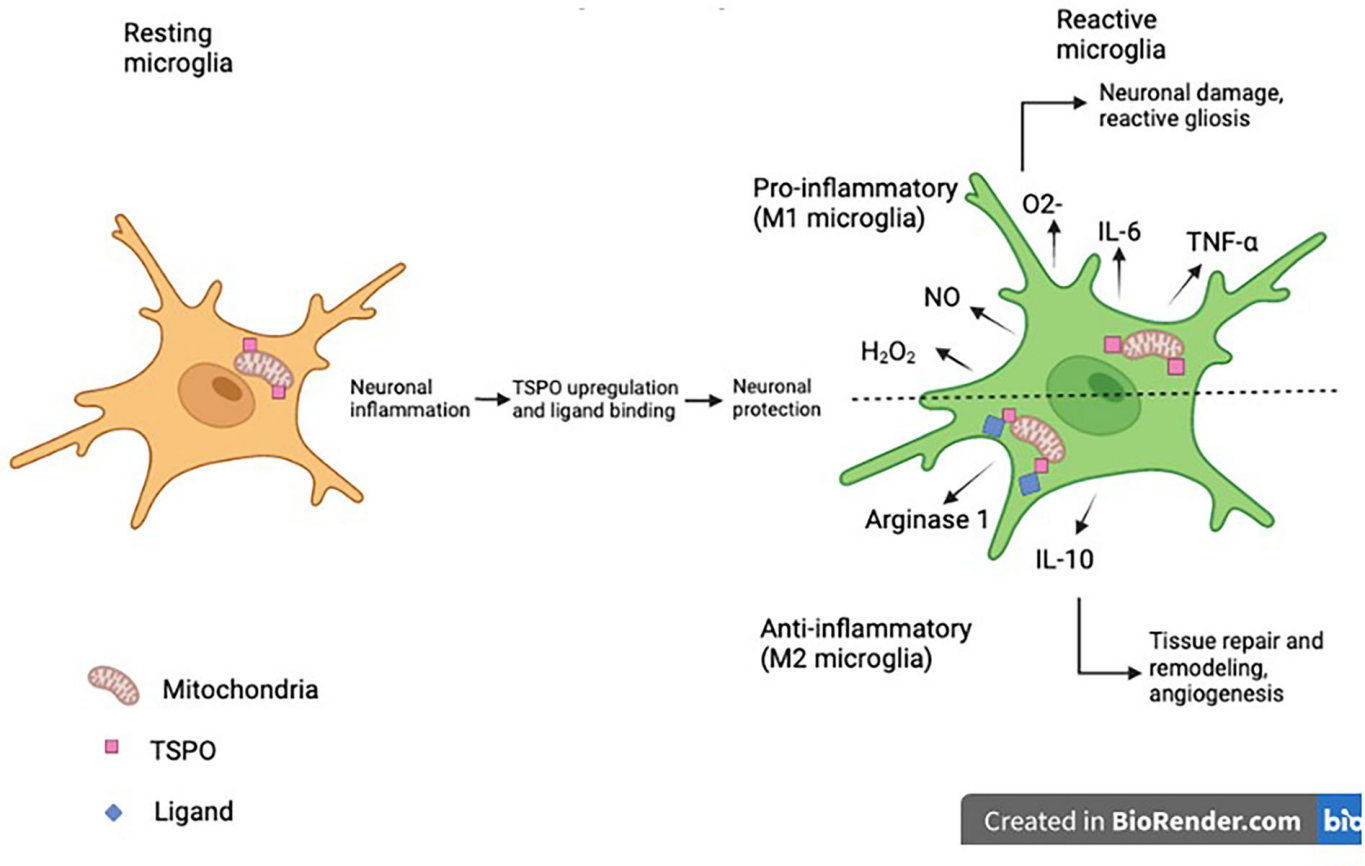
Mechanism underlying the translocator protein (TSPO) binding

Various types of PET imaging have been developed since its discovery. One of the most widely used pharmaceuticals is the positron labeled 2‐fluoro‐2‐deoxy‐D‐glucose (FDG).^21^ The image created by the PET scan is based upon the Warburg effect which states that high metabolic activity within cancer cells is due to increased glucose utilization in order to sustain continuous cell growth and division (Figure 2).^24^ Due to this phenotypic property, the positron emitting ^18^fluorine (^18^F) linked to the antimetabolite glucose analog 2‐deoxy‐D‐glucose (2‐DG) accumulates as a tracer in the region of the tumor making imaging possible.^21^ The FDG‐PET/CT has shown accurate staging of nonsmall cell lung cancers, anatomical and functional information of nonoperative head and neck cancer patients following radiation and chemotherapy, improved diagnostic accuracy for recurrent and metastasized thyroid cancers, and an enhanced therapeutic ratio when incorporated with radiation treatment planning.^25^ FDG‐positron emission mammography along with dedicated CT is recommended for breast cancer screening which can detect tumors as small as 1 mm.^25^ However, interpretation of the results from a FDG‐PET scan is made with caution as extraneous tissues like the brain, liver, and dense breast tissue may also have high FDG uptake and possess certain intrinsic characteristics that obscure the image. For example, brown fat serves the purpose of producing heat via its numerous mitochondria; the increased metabolic activity occurring within this tissue could induce FDG uptake and highlight areas that are actually cytologically normal.^26^ New tracers are being developed that could potentially have greater sensitivity and specificity than the existing tracers.^2^, ^7^ One area of growth is individualized scans using tracers that are tailored to patients through gene profiling of their tumors.^21^ Using specific tracers based on the circumstances of the patient may decrease background signal on the image and thus, create a higher quality image without any obscuration; this provides patients with the best diagnostic accuracy in the context of their clinical situation.^9^, ^21^ Specific radiotracers such as ^11^C‐ and ^18^F‐choline, ^11^C‐methionine, ^18^F‐ DOPA, ^68^Ga‐DOTA‐somatostatin analogs, ^68^Ga‐ligand‐prostate specific membrane antigen (PSMA), ^18^F‐PSMA, and ^68^Ga‐fibroblast activation protein inhibitor (FAPI), are being clinically tested for the use of differentiating non‐neoplastic etiologies such as infection, noninfectious inflammation, and tissues with normally increased physiological uptake (e.g., the central nervous system).^9^ Typically, these tracers have radioactive properties that allow for the progressive decay through the exposure to positron emission. The most widely used radiotracer within the clinical setting is F‐fluorodeoxyglucose (FDG) which is essentially a mixture of fluorine‐18 and deoxyglucose. The reasoning behind the popularity of this tracer is due to the increased rate of glucose metabolism displayed by cancer cells. For instance, when deoxyglucose is labeled with 18‐flourine (18F) which is a positron emitting radionuclide, the cancer cells become more easily detectable using PET. After 18F‐FDG is transported into these cells, it is phosphorylated to FDG‐6‐phosphate (FDG‐6‐P) via hexokinase or glucokinase; instead of entering the typical metabolic pathways of glucose, this compound exhibits “metabolic trapping” or accumulation within neoplastic cells due to the presence of fluorine, instead of the typical hydroxyl group in glucose, at the C‐2 position of the ring structure.^27^ This trapping is what provides the basis of the use of this specific PET radiotracers within the field of oncology. Typically, PET scans are better suited for identifying early stages of cancer, but they are also commonly used for evaluation of recurrence, especially for colorectal and lung cancers, melanomas, and lymphomas.^21^, ^25^, ^28^, ^29^ A major development in this area is the transition from whole body PET scans to total body PET scans with the completion of the EXPLORER in 2019‐ the first total body PET scanner.^30^ Whole body PET scans are typically used to identify metastasis in melanomas but there are several limitations including its restricted field of view (FOV) which is the main contributing factor to the poor sensitivity of this imaging modality.^30^, ^31^ Total body PET scans mitigate the lack of sensitivity seen with whole body PETs by utilizing a cylindrical scanner to encompass the entire body within its field of view, creating a 40 fold increase in the sensitivity.^31^ The area of PET imaging is expanding due to the growing accessibility of equipment necessary to conduct these studies. New developments are constantly being made to increase the efficacy and broaden the utility of PET scans. PET instrumentation has undergone significant advancements in hardware, reconstruction methods, implementation of time of flight (TOF) technique in clinical practice, improved CT component, and introduction of PET/MR.^32^ The sensitivity and specificity of 3D PET imaging systems has been improved with the removal of interplane septa that physically separates detector rings along the axial dimension and the use of new detector material that allows the generation of higher spatial resolution images.^9^, ^21^, ^33^ High density and low decay time (time needed to decrease detector's light output pulse to 36.7% of its maximum‐amplitude value) PET detector materials like bismuth germanate oxide (Bi4Ge3O12 or BGO) and cerium‐doped gadolinium oxyorthosilicate (Gd2SiO5 (Ce), or GSO), together with a newer and widely used material, cerium‐doped lutetium oxyorthosilicate (Lu2SiO5 (Ce) or LSO), are indispensable for high photon counting rates and high resolution in PET imaging.^31^ These PET detectors are also essential for time of flight (TOF) PET imaging (Δt is the time difference in the detection times of two photons from the same coincidence event). TOF imaging demonstrates high image quality as smaller Δt results in smaller line of response (LOR) where annihilation is likely to occur, causing improved signal to background ratio.^9^, ^34^ The technological advances in 3D iterative reconstruction has enhanced the performance of PET imaging related to reconstruction time, scatter correction, incorporation of CT‐attenuation maps, random events, spatial system response and dead time.^31^ There have been several new advancements within radiation oncology in terms of utilizing PET scans in conjunction with certain tracers in order to identify and stage several types of cancer. For example, 18 kDa translocator protein (TSPO) is expressed in glioblastoma and several other neurodegenerative disorders. TSPO PET is an imaging modality that utilizes tracer specific to the TSPO protein in order to identify foci of inflammation within the brain and can lead to an earlier diagnosis of the malignancy (Figure 3).^35^ Tissue specific enzyme production is also helpful in diagnosing prostate malignancies. PSMA is an enzyme produced by the body that is highly specific to the prostate. PSMA PET scans that utilize radioactive tracer labeled with galium or fluorine targeting this specific enzyme have been shown to have high sensitivity when used for prostate cancer screening, even when PSA levels are low. Enhanced amino acid synthesis is one of the indicator of extensive proliferation of cancer cells, therefore PET imaging agent L‐methyl‐11C‐methionine (11‐C‐MET) which measures methionine accumulation has been used in breast cancer patients, glioma, and leptomeningeal metastases.^36^, ^37^, ^38^, ^39^ Due to the rapid metabolism and short half‐life of 11‐C‐MET has limited the clinical utility and has posed time constraint on PET‐imaging, compromising the image acquisition and quality. Alternatively, fluorine‐18‐labeled amino acids with long half‐life, such as L‐3, 4‐dihydroxy‐6‐18F‐fluoro‐phenylalanine (18F‐DOPA), O‐18F‐fluoromethyl‐D‐tyrosine (18F‐FMT), O‐(2‐18F‐fluoroethyl)‐L‐tyrosine (18F‐FET), and anti‐1‐amino‐3‐18F‐fluorocyclobutane‐1‐carboxylic acid (18F‐FACBC) has been used clinically for PET‐imaging of variety of tumor types. 18F‐DOPA PET detected glioblastoma with high accuracy and predicted progression free survival. Studies indicated that 18F‐FET PET/CT added diagnostic information in brain stem and spinal cord glioma. 18F‐FACBC has been shown to be a better PET imaging agent for prostrate tumor due to its slow metabolism that prevents its rapid accumulation in the urinary bladder.^40^ Thymidine analogs such as 11C‐thymidine, 3′‐deoxy‐3‐18fluorothymidine (18F‐FLT), and 1‐(2′‐deoxy‐2′‐fluoro‐1‐β‐D‐arabinofuranosyl)‐thymine (FMAU) imaging are used to measure the proliferation of tumor cells, targeting the thymidine uptake during DNA synthesis, suggesting the prognosis and aggressiveness of a tumor.^39^, ^41^ 18F‐labeled nitroimidazoles and Cu‐labeled diacetyl‐bis (N4‐methylthiosemicarbazone) analogs PET‐imaging has been utilized to measure hypoxia, implicating in determining the resistance of tumor to radio‐chemo‐therapy.^42^ Therapeutic resistance has also been evaluated using various PET‐based imaging biomarkers including sex hormone receptors, oncogenic receptors, and angiogenic factors.^39^ Further, PET‐apoptosis imaging using 18F‐ML‐10 from the Aposense and 18F‐CP18 (radiolabelled caspase 3 substrate) evaluates the extent of apoptosis‐induced cell membrane asymmetry and acidification for assessing the therapeutic response in cancer patients.^43^ F‐FDG PET scans have proven to have utility in head and neck cancers, lung cancers, and gastrointestinal cancers including esophageal, rectal, anal, and pancreatic cancers. F‐FDG PET scans can help with staging and may indicate prognosis by identifying areas of malignancy and potential lymph node involvement to help determine the extent of the cancer and course of treatment. In addition, the scans can be used for target volume delineation, assessing the shape and size of the area to get as accurate a representation of the tumor as possible. This data is important in planning for radiation treatment so that doses can be adjusted in order to be as efficacious as possible while also limiting the amount of radiation the patient is exposed to. 3D iterative reconstruction algorithms like point‐spread function (PSF) has accelerated the prediction of input signal during reconstruction and filtered back projection which has been used to reconstruct images in addition to Shepp‐Vardi algorithm, a statistical likelihood‐based iterative expectation‐maximization algorithm. Hybrid PET‐CT systems use CT‐based attenuation correction for low‐noise attenuation maps, fast data acquisition, and elimination of bias from emission contamination of post injection transmission. Recently introduced hybrid PET‐MR system provide capabilities of both PET and MR imaging modalities simultaneously, however improvements in the instrumentation are still underway to reduce cross‐interference of PET and MR signals. Furthermore, innovative PET technologies like carbon ion beam therapy monitoring with INSIDE in‐beam PET scanning, and so forth. are emerging for cancer treatment.^44^ Several clinical limitations of PET‐based imaging are the associated logistical constrains, probability of variability between measurements, lack of training in computational and machine learning/ deep learning methodologies, and unsuitable statistical procedures to collect repeatable and reproducible large data‐set.^45^ Involvement of multi‐center and multi‐disciplinary studies will over some of the limitations besides generating cross‐validation algorithms and predictive models.

### 2.2 X‐ray computed tomography

Computed tomography is another method of imaging that can be utilized to diagnose cancer. CT has been effectively used for the screening of colon, lung, head and neck, breast cancers and so forth. with an accurate spatial and temporal tumor imaging, aiding in follow‐up biopsy procedures, surgery, and radio‐ chemo‐therapy.^46^, ^47^ Several instrumentation advancements such as scan speed, dual energy, iterative reconstruction, low kilovolt, perfusion imaging, and radiation dose reduction have accentuated the clinical utility of CT‐based tumor imaging.^48^ The spiral multi‐detector CT with multi‐fan measurement technique has resulted in improved spatial resolution, and has eliminated artifacts via high‐end reconstruction and noise reduction algorithms. Photon counting (counting incoming photons and measuring their energy) and artificial intelligence has also enabled a high resolution CT image reconstruction, reduction in radiation dose, and artifacts. The CT scan is frequently used in addition to the PET scan to provide precise anatomical localization of the lesions revealed by PET.^24^, ^49^ The PET scan relies on the biochemical reactions taking place within the cells to form an image with low spatial resolution, while the CT scan creates an image with high spatial resolution that delineates the structural and morphological characteristics of the tumor.^24^ Moreover, the concurrent CT scan corrects for the inherent attenuation seen with PET scans, helping to increase both the sensitivity and specificity of the image.^49^ In a study conducted by Shawky et al., comparing PET/CT to CT alone in detection of breast cancer, the sensitivity of PET/CT examinations was 100%, specificity was 95.4%, PPV was 88.9% and NPV was 100% while the sensitivity of CT alone was 81.2%, specificity was 90%, PPV was 76.4% and NPV was 93%.^50^, ^51^ However, it is important to be aware of the fact that when performing an add‐on CT, there is a high probability of artifacts obscuring the superimposed PET, especially when using contrast agents.^24^ CT scans are also frequently performed with SPECT scans. SPECT/CT hybrid imaging is typically used for detecting metastasis in cancers with increased skeletal affinity such as breast, prostate, and lung carcinomas.^24^ There are two types of SPECT/CT that are used in today's practice: targeted and whole body. In a study conducted by Rager et al., whole body SPECT/CT imaging particularly improved detection of extra‐axial skeletal lesions with whole body imaging actually changing the diagnosis in 5.7% of study participants that were initially imaged with targeted SPECT/CT.

### 2.3 Magnetic resonance spectroscopy

Magnetic resonance spectroscopy is a widely used form of imaging used for the diagnosis of cancer. It is most widely used in the diagnosis of brain tumors, but has more recently been applied in the diagnosis of pancreatic, prostate, breast, cervical, and gastrointestinal cancers. MRS differs from the conventional MRI in that signals from compounds such as carbon, hydrogen, creatinine, lactate, and N‐acetylaspartate are measured rather than signals from water.^26^, ^52^ MRS is based on the concept of Larmor frequencies which demonstrates that protons in different compounds move at different frequencies based on the distribution of surrounding electrons. When a magnetic force is applied externally, the electrons generate a magnetic field since they are charged particles and proportionally shifts the frequency of the molecule. These changes in frequency are measured which can provide data regarding the composition of the area being imaged.^53^ Rather than providing images of soft tissues, MRS quantifies various compounds like lactate, phosphocreatine (PCr), nucleotide triphosphate (NTP), phosphate monoesters, and inorganic phosphates; it also functions to determine the presence and varying amounts of these compounds in different target tissues which can potentially signify an abnormality.^54^ The presence of lactate at the long time of echo (TE) when analyzing the brain indicates a pathological irregularity, suggesting the presence of a malignant lesion.^52^ MRS detects metabolic changes in tumors such as total choline (Cho) levels and ratios with other metabolites which may suggest the proliferative potential and aggressiveness of the cancer tissue.^55^ MRS can also be utilized to help differentiate between tumor recurrences and tumor necrosis by looking at ratios of such metabolites. Single 1 cm × 1 cm × 1 cm voxels are placed throughout a lesion to occupy the space of the lesion and peak intensities of the metabolite are measured in each voxel. The ratios of the metabolites can provide information regarding the lesion that is being examined. For example, elevations in Cho/NAA and Cho/Cr ratios in combination with a decreased NAA/Cr ratio signify that the lesion in question is more likely to be a tumor recurrence while NAA/Cr ratios appear higher in radiation injury and tumor necrosis.^56^ N‐acetyl aspartate (NAA) is used to distinguish between low and high grade astrocytomas and gliomas, while phosphocholine levels are used to detect malignant breast cancer cells.^55^ Techniques such as hyperpolarized MRI facilitate the detection and characterization of the particular types of cancer. The hyperpolarized 13C‐labeled pyruvate infusion to monitor glycolysis is the most commonly used substrate. Following the administration of 13C‐labeled pyruvate, the rate of hyperpolarized 13C lactate flux is measured which typically increases with cancer progression and decreases with treatment initiation.^55^ More recent studies have also demonstrated the beneficial impact of this imaging method on determining the pH of tissues. Because many pathologic conditions are associated with specific pH alterations, monitoring pH within the tissue may potentially be helpful as a marker for treatment efficacy.^57^ In addition to the hyperpolarized MRI, is the chemical exchange saturation transfer (CEST) which is a new contrast enhancement technique that provides high resolution images of molecules and macromolecules that are capable of exchanging protons with surrounding water molecules.^55^ The enhanced sensitivity of this technique allows for the detection of minute concentrations of cellular components. Amide proton transfer (APT) imaging is one of the most popular CEST‐based methods, and it works via the exchange between protons of water and those of amide groups within endogenous peptides. A very tight relationship exists between the aggressiveness of the cancer and the accumulation of defective proteins, which is the reason why this technique is particularly useful in diagnosing high‐grade brain malignancies for instance, as the peptide levels are substantially elevated.^58^, ^59^ In a study comparing rats implanted with either 9L gliosarcoma cells or human glioblastoma cells.^24^, ^49^ T magnetic field strength imaging was used to determine the signal intensities of these tumors.^60^ The study concluded that the APT‐weighted images demonstrated an increased signal intensity compared to conventional transfer techniques which was beneficial in identifying specific anatomical regions and mapping out distributions of the tumor. Overall, the mechanism by which APT functions at a protein level is very unique and may have potential applications within the clinical setting in the future. Whole‐body MRI (WB‐MRI) has been implicated in the management of cancer patients, and several studies suggest the use of WB‐MRI for screening of malignant tumors in asymptomatic subjects.^61^ These technological advances in MRI‐based imaging have played a substantial role in early diagnosis and therapeutic management of cancer patients.

## 3 MOLECULAR DIAGNOSTIC TECHNIQUES

### 3.1 Biomarkers using multi‐parameters analyzed by flow cytometry

Multi‐parameter flow cytometry can detect simultaneously numerous cell markers present on the cell surface as well as intracellular antigens, besides cell size, texture and DNA content (related to ploidy) that has been gainfully applied in the diagnosis as well as prognosis of all malignancies. The technique is objective and allows analysis at the single cell level on a large number of sample size (cell number) that makes the technique reliable and reproducible and more accurate than immunohistochemistry in a number of liquid cancers including hairy cell leukemia. Since many molecular and cytogenetic changes are associated with immunophenotypic characteristics, employing appropriate combinations of fluorescent labeled antibodies and other probes is powerful approach for the diagnosis of hematological and other malignancies. For example detection of hairy cell leukemia can be easily accomplished by using BRAF V600E mutation specific antibody. FCM can be used to diagnose several hematological malignancies, like acute lymphoblastic leukemia (ALL) and detect clonality in B or T proliferations, making it an indispensable technique in the detection and staging of hematological malignancies. New scoring systems like EGIL and Matutes scoring have been developed that uses a panel of different markers to determine the normal counterpart of tumor cells and in the immunophenotyping of chronic lymphocytic leukemia (CLL).^30^, ^31^

### 3.2 Oncotype Dx

A diagnostic or more appropriately a prognosis and management aiding tools have been recently developed that provides a personalized risk assessment and helps the therapy administering oncologist in the selection of an appropriate therapeutic strategy. These are algorithm based analysis of comprehensive data on clinical, radiological, pathologic, genotypic, and phenotypic markers. These were developed originally to facilitate the management of breast cancer patients following conservative breast cancer surgery based on the risk analysis of relapse linked to the genomic, transcriptomic and proteomic analysis. One of them is Mammaprint that is based only on a 70 breast cancer gene signature irrespective of the clinic‐pathological data, while another one; Oncotype Dx Breast is based on a 21 gene breast cancer signature combined with clinical and pathological data. Currently, Oncotype Dx has been developed for the management of other cancers as well.^32^, ^44^ More recently, assays based on the multi‐gene status of tumors have been developed that helps in the assessment of relapse potential based on the biological behavior of tumors.

### 3.3 Synthetic biomarkers

Synthetic biomarkers are a novel class of cancer diagnostic tool that uses a biosensor sensor placed inside the body to identify phenotypic changes at an early stage of the tumor and amplify this cancer related signals to a very high level that can be easily quantified. This approach is developed based on significant advances made in the areas of chemistry, synthetic biology and cell engineering and is for more sensitive than methods that analyze biomarkers that shed into the body fluids.^46^

### 3.4 Exosomes

Exosomes are extracellular vesicles secreted from many body cells that carry metabolites, RNAs (mRNA, miRNA, long non coding RNA), DNAs (mtDNA, ssDNA, dsDNA) and lipids from the cells in which they were generated and contribute to the intercellular communication.^47^ Due to their stable nature, they are easily accessible as they are found in the bodily fluids like urine, plasma, saliva, and breast milk and bear a relationship with the cells of origin. They have been exploited as a biomarker for the early detection of cancer as their contents reflect the genotypic and/or phenotypic (aberrant proteins) alterations of the cancer cells they originated.^62^ Due to their minimally invasive nature of access, they have an edge over the highly surgical tissue biopsy.^63^

### 3.5 Nano technology

Due to their small size, biosafety, better loading of diagnostic probe, and physical properties nanoparticles have been gainfully employed in various imaging based cancer diagnostics. Quantum dots that emit fluorescence in the near infrared region and have better tissue penetration has been used in combination with tumor specific biological probes (peptides, antibodies and other small molecules) for improved imaging, while silver‐rich Ag2Te quantum dots that provides a better spatial resolution has also been used for tumor imaging.^64^, ^65^ Similarly, gold nano particles a good contrast agent with better biocompatibility and nanoshells have also been used for imaging of tumor tissue for early detection of malignancy.^66^ Nanotechnology has also been used to assess the tumor microenvironment be exploiting the typical response of fluorescent nanoprobes to pH that helps in the detection of fibroblast activated protein‐α in the tumor‐associated fibroblasts.^67^ The recent development of MXene based biosensors with high conductivity and superior fluorescent, optical, and plasmonic properties have been found be promising for the detection of cancer biomarkers due to their high sensitivity (femtomolar range for detection).^26^

### 3.6 Fluorescence in situ hybridization (FISH)

Assessment of chromosomal changes acquired by the cancer cells plays an important role in the diagnosis and therapy of various malignancies and particularly in hematological malignancies where they are more prevalent. Classical cytogenetic techniques based on various banding techniques require dividing cells to detect cryptic rearrangements by analyzing the metaphase chromosomes. With the advent of various molecular cytogenetic techniques like fluorescence in situ hybridization (FISH), identification of complex and cryptic chromosomal abnormalities such as exchanges and gene rearrangements, amplifications, and deletions can be accomplished at the single cell level even in the interphase cells and in frozen sections. These techniques make use of probes that identify specific nucleic acid sequences (DNA or RNA) by hybridizing with the commensurate sequences and report with the use of fluorescent molecules tagged to the probes. With the proper selection of a combination of probes and fluorescent reporters simultaneous hybridization of several loci can be accomplished resulting in multiplex FISH called the MFISH, spectral karyotyping (SKY), combined ratio labeling (COBRA).^68^, ^69^ Spectral karyotyping (SKY) is a multi‐chromosomal painting assay that uses 24 colors allowing the simultaneous visualization of all human chromosomes so that complex chromosomal rearrangements and extra‐chromosomal structures and recurrent chromosomal aberrations can be analyzed. The SKY has been successfully used for analyzing various tumors such as hematological malignancies, sarcomas, carcinomas and brain tumors aiding the clinical diagnosis and prognostic assessment.^70^ By comparing the hybridization pattern of cancer cells with the normal cells comparative genomic hybridization (CGH), can identify chromosome losses and gains in tumor cells without prior knowledge about the chromosomal loci involved. Moreover, when combined with immunocytochemistry, these in situ hybridization techniques can provide information on the relationship between gene activity at the DNA and mRNA levels and the topographic information.^71^, ^72^, ^73^

### 3.7 ctDNA and liquid biopsy

Cell free DNA (cfDNA) found in the blood circulation and related to the tumor is widely referred to as circulating tumor DNA (ctDNA). Using next‐generation sequencing (NGS) coupled with other advances in the analysis of ctDNA an association of ctDNA with tumor stage, tumor burden, prognosis and response to therapy has been well‐established.^74^ Recent studies showing genetic and epigenetic changes in ctDNA have shown the potential of ctDNA in the early detection of cancers that can improve the clinical management of cancer patients.^75^ More recently minimal molecular disease (MRD) detected using ctDNA immediately after a course of therapy namely, surgery, chemotherapy and so forth or a molecular relapse generally detected by ctDNA after a time gap following therapy has been correlated with high rate of recurrence including metastatic recurrence detected by radiological and other imaging modalities.^76^, ^77^, ^78^

### 3.8 Liquid biopsy

Liquid biopsy is one of the novel and nontraditional diagnostic concept that uses minimally invasive procedure to obtain information on the molecular signatures of solid malignancies using body fluids like blood, saliva, and urine. It has been exploited as a powerful tool in personalized medicine, as it can be used to monitor the progress of the disease in real time. Liquid biopsy supports the analysis of Circulating Tumor Cells (CTCs), ctDNA, circulating (or cell free) miRNAs (ctmiRNAs) and extracellular vehicles (EVs) that has greatly facilitated the early detection, prognosis, and design of tumor specific therapy thereby improving the overall management of cancer. Currently, liquid biopsy is one of the arms of many clinical trials that has improved our understanding of the metastatic process based on mechanistic and translational studies using CTC derived cell‐lines and explants (CDx).^79^, ^80^

### 3.9 Volatile organic compound (VOC) analysis

Breath volatile organic compound (VOC) analysis is one of the techniques for early detection of lung cancer that aims at analyzing various molecular signatures from the exhaled breath of lung cancer patients using gas chromatography mass spectrometry (GC‐MS) and sensor technologies that could be used as biomarkers of lung cancer. More than 1000 VOCs have been identified from the human breath that depends on a number of factors related to the physiological conditions, environmental factors, age and gender, comorbidity, disease status and staging. Although VOCs found across many clinical studies does not overlap among different malignacies, nearly 15 molecules have been found consistently in 4 to 10 studies. These include ethanol, acetone, isoprene, pentane, hexanol, toluene, benzene, ethylbenzene, heptane, 2‐butanone, styrene, pentanal, butanol, and so forth. While results from studies using various sensor technologies have been encouraging, findings from preclinical and clinical studies with mass spectrometry have been so far equivocal in generating a consistent molecular signature patterns related to lung malignancy.^81^

### 3.10 Long noncoding RNAs (lncRNAs)

It is now well established that a large amount of genomic mutations associated with cancer reside in nuclear DNA regions that do not encode proteins, but are transcribed into long noncoding RNAs (lncRNAs). Application of next‐generation sequencing has revealed a strong association of aberrant expression many lncRNAs with different cancer types and linked with malignant transformation and playing important roles in proliferation, survival, migration, or genomic stability.^82^, ^83^

## 4 IMMUNOHISTOCHEMISTRY OTHER MOLECULAR DIAGNOSTIC TECHNIQUES

A vast array of molecular biology techniques has been developed for cancer diagnosis and subtyping, such as immunohistochemistry, immunofluorescence, flow cytometry, and DNA and RNA‐based hybridization/sequencing approaches. Immunohistochemistry (IHC) is a common method that relies on the expression and upregulation of tumor specific antigens from the cancerous cells in order to make a diagnosis.^84^ Monoclonal or polyclonal antibodies are tagged with fluorescent dyes to directly target these antigens, and then the tissue in question is viewed under a microscope to identify the areas marked by the fluorescence.^84^ This method of imaging is growing in popularity due to its specificity as the antibodies can be altered in order to target antigens expressed by very specific forms of cancer.^84^ For example, if a neuroendocrine tumor is suspected, IHC can be performed using antibodies that target the chromogranin antigen; a positive result indicates the presence of neuroendocrine cells within the tumor, and the brightness of the fluorescence approximates what portion of the tumor is composed of these cells.^85^ The most imperative application of IHC in oncology today is specifically for the diagnosis of small round blue cell tumors and lymphomas as they identify the presence of specific “cluster of differentiation” (CD) markers which are used to identify specific cancers within these categories.^85^ IHC is also used as a prognostic indicator for specific cancers such as breast cancer by identifying the presence of HER2/neu protein, estrogen and progesterone receptors, and markers of proliferation such as Ki 67. As IHC becomes a more prominent method of cancer diagnosis, its use is expanding for the detection of micrometastasis to lymph nodes.^85^

Molecular methods of cancer diagnosis are gaining traction in the field of oncology. One of the most significant techniques in this category is the microarray which allows for the study of DNA, RNA, and proteins.^85^, ^86^ In the DNA microarray‐based technique for the analysis of global gene expression, RNA is obtained and reverse transcribed using fluorescently labeled nucleotides resulting in labeled cDNA.^85^, ^87^ Hybridization of the cDNA with the preexisting probes in the microarray followed by scanning and analysis, provides important molecular information which can have significant diagnostic and prognostic implications.^85^ For example, cDNA hybridization has been used to obtain prognostic information in patients with neuroblastoma. A cDNA microarray consisting of 5340 genes was obtained from primary neuroblastomas. These genes that were incorporated into the microarray were carefully selected as these specific sequences code for a protein that had an effect on the severity and prognosis of the malignancy. By utilizing this cDNA microarray to identify what genes were present, they were able to attain a prognostic outcome prediction that was accurate 88.5% of the time in the subjects that were studied. The microarray is advantageous in that it provides very specific objective information regarding a tumor.^87^, ^88^ It has also proven to be useful in patients with metastatic carcinomas by identifying primary sites and highlighting small variations in gene expression within the tumor.^74^, ^87^ However, there are several limitations to microarrays which prevent it from being used more frequently. First, there are not enough objective tumor markers to warrant the widespread usage of microarrays in all contexts.^87^ Additionally, since the efficacy of microarrays is dependent on the information existing within the reference databases, it is not always feasible to use, especially with rare conditions that do not have enough accessible information.^87^, ^89^ Finally, it is unable to detect tumors in their early stages as this method requires a significant quantity of tumor cells to be efficacious.^87^ Due to the constant acquisition of new knowledge in this field, these limitations are being addressed with time. The state‐of‐the‐art technique, next generation sequencing (NGS), aids in unraveling the cancer diversity, specific recurring mutations, and novel molecular targets for therapy.^90^, ^91^, ^92^ NGS allows multigene analysis and mutation status of cancer‐causing genes, with excellent technical performance and decreased rates of false results. Robust technical expertise is warranted for precise interpretation of high‐throughput data sets.

**FIGURE 4:**
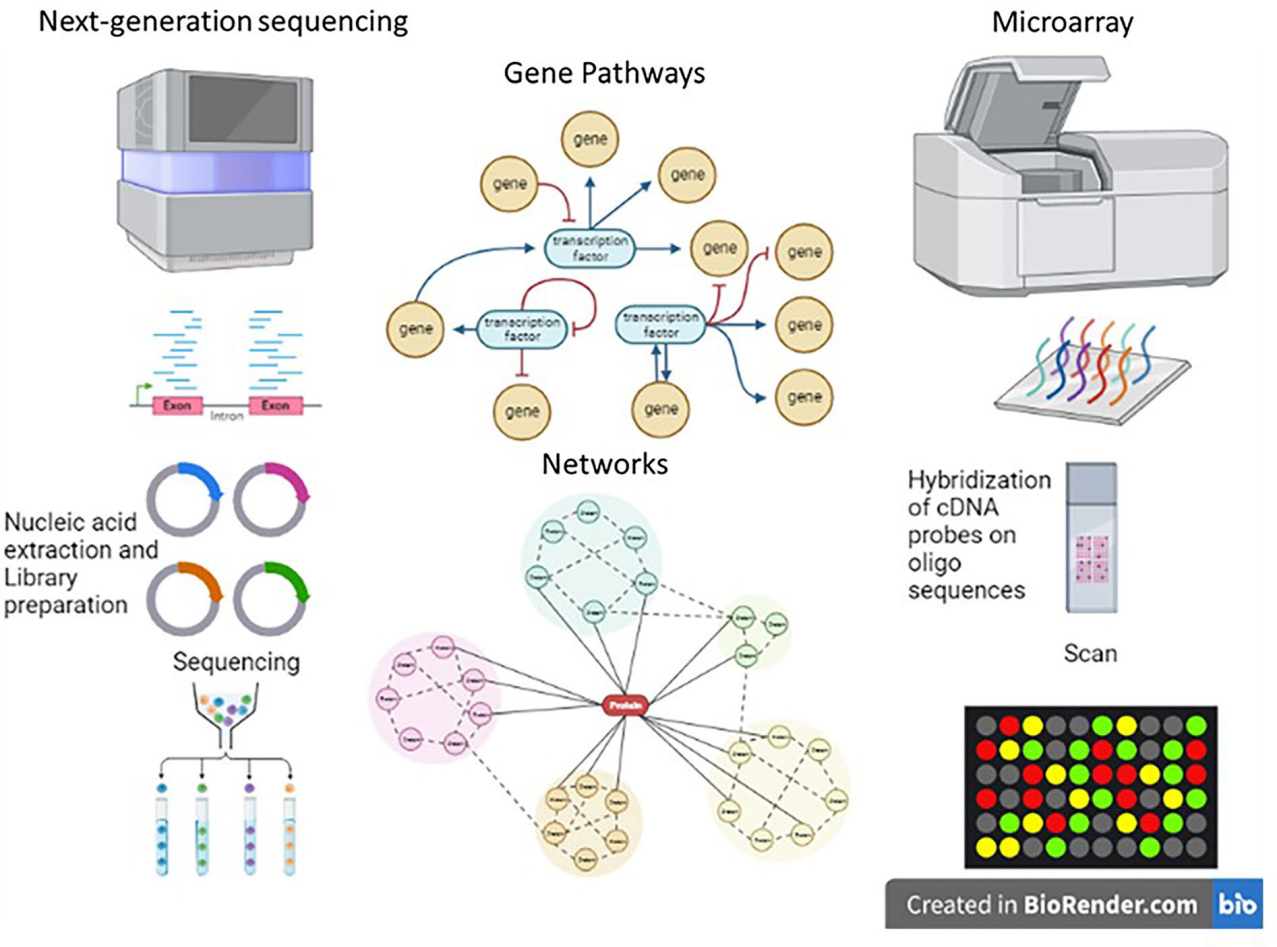
Next‐generation sequencing and microarray as advanced tools for cancer diagnostics and personalized medicine

As we continue to study various cancers, begin to identify new tumor markers, and expand the reference database, molecular techniques such as the microarray and NGS will become more prominent methods used for cancer diagnosis (Figure 4). Recently, NGS has been demonstrated to detect point mutations and insertions in BRCA1/ BRCA2 genes highlighting the importance of NGS in predicting the risk of developing hereditary breast cancer, besides reducing time and cost.^93^ In addition to mutational screening RNA Seq or single cell RNA Seq has accelerated the methods in cancer diagnostics.^94^

### 4.1 Low‐cost diagnostics and treatments

Diagnostic tools are imperative for the prevention, identification, and treatment of various cancers and other disease states. However, the high cost of diagnostic modalities frequently hinders their utilization by specific patient populations.^95^ Many of the traditional diagnostic imaging techniques cannot be conveniently used by patients of low‐income status and those that live in low‐ and middle‐income countries (LMICs) with resource‐poor settings, especially due to high expense and lack of substantial medical infrastructure.^96^ A great number of individuals globally live in resource‐limited areas and therefore, have limited access to traditional diagnostic tools. This is a major concern, especially because an increase in cancer rates seems to be affecting developing countries disproportionately; cancer diagnosis are occurring at later stages with an increase in morbidity and mortality rates, and also are associated with a more expensive treatment plan.^96^, ^97^, ^98^ This discrepancy has led to an interest in developing more low‐cost diagnostic tools for a large fraction of the global population with minimal resources and underdeveloped healthcare settings.

For example, cervical cancer is the third most prevalent cancer affecting women globally, with a majority of cervical cancer deaths occurring in LMICs.^96^, ^99^ When targeting the issue of cervical cancer prevention, it is important to understand that there is an identifiable precancerous stage, also known as cervical dysplasia, which develops from oncogenic strains of human papilloma virus (HPV). This provides a longer period of time to recognize these precancerous squamous intraepithelial lesions to potentially prevent the progression to invasive stages. In 2018, there were approximately 569 000 new cases of cervical cancer and 311 000 deaths worldwide; around 84% of the new cases and 87% to 90% of the deaths occurred in LMICs.^100^ Due to the high prevalence especially in LMICs, the World Health Organization (WHO) launched a global strategy in the attempt to eliminate cervical cancer as a major public health problem.^101^ Their goal consists of 90% HPV vaccination coverage, 70% screening coverage, and 90% access to treatment options by 2030.

Traditional colposcopy plays an important role in the primary screening of cervical cancer by guiding cervical biopsies, but has a limited use in LMICs. Fortunately, other alternative diagnostic tools such as artificial intelligence (AI)‐guided colposcopies are starting to gain popularity, especially in LMICs in order to improve cervical cancer screening and prevent the development of cervical intraepithelial neoplasia (CIN).^102^ AI mechanisms like the deep learning‐based algorithms are beneficial since they can recognize specific features of intraepithelial lesions using the colposcopy images, which can then be incorporated directly into the digital colposcopy.^102^ This screening method has the vast potential to increase the accuracy of colposcopy with digital imaging. In addition, the availability of AI technology could minimize the discrepancy in colposcopy‐based diagnostics between tertiary hospitals and primary care hospitals.^102^ Therefore, the integration of AI‐guided digital colposcopy may have a significant role in improving the diagnostic performance of colposcopy, especially in LMICs in the future.^103^ In other words, the use of AI‐guided digital colposcopy may enhance the quality of medical care, provide a cost‐saving cervical cancer screening methodology, and allow equal access to diagnostic tools across all patient populations.^102^ A retrospective study in the Cervical Pathology Diagnostic Unit of the Department of Obstetrics and Gynecology at Wroclaw Medical University in Poland analyzed 48 colposcopy examinations in patients that had an abnormal clinical impression of the cervix after speculum examination, abnormal PAP smear, or abnormal PAP smear with a positive high‐risk HPV test. Every patient had their cervix examined manually by a gynecologist and an AI‐based algorithm called VisualcheckTM that analzyed images acquired from the procedure in order to determine the presence of ≥CIN2+ cervical pathologies. The difference between the results of the AI‐based algorithm and the gynecologist was statistically significant with *p* equaling .003. While the gynecologist classified 43 of the 48 exams as abnormal, the AI‐based algorithm classified only 28 out of 48 images to be abnormal; out of the 43 exams considered to be abnormal by the gynecologist, only 18 demonstrated true findings of CIN2+. The statistical results of this study illustrates congruence between the AI‐based algorithm results and the clinical assessment when differentiating between a normal or abnormal cervix in 60% of the cases. Additionally, the PPV, NPV, sensitivity, and specificity of the AI‐based algorithm's detection of CIN2+ was 42.9%, 70%, 66.7%, and 46.7%, respectively. However, the PPV, NPV, sensitivity, and specificity of the gynecologist's clinical assessment was 41.8%, 100%, 100%, and 16.7%, respectively. Collectively, the results demonstrate that experienced gynecologists may over diagnose cervical pathologies, and although there is a reduction of false negative results when a clinical assessment is performed by a gynecologist, that benefit comes with the associated risk of a low specificity. On the other hand, the AI‐based technique was able to detect pathologies similar to that of the gynecologist with a PPV, sensitivity, and specificity of 42.9%, 66.7%, and 46.7%, respectively. Although the AI‐based method displayed a much lower sensitivity compared to the gynecologist's assessment, the specificity was significantly greater. A major limitation of the study is the small sample size, but these results still support the potential use of AI has in expanding he human diagnostic ability within the oncologic setting. As this technique is further explored, especially with the application of AI in the clinical setting recently gaining a significant amount of popularity, an improvement in the efficiency of cancer diagnosis, a decrease in the workload of physicians, and an enhancement in the management plan of patients is likely.

In regards to the current treatment regimen for those with a positive cervical cancer screening test, the WHO has recommended the use of cryotherapy as a first line therapy. However, because there is limited access to traditional cryotherapy interventions in LMICs, an alternative cost‐effective cryotherapy device called CryoPop® was developed by Jhpiego, a nonprofit organization affiliated with Johns Hopkins University that focuses on women's health dedicated, and the Johns Hopkins Center for Bioengineering Innovation and Design.^96^ Most traditional cryotherapy interventions utilize nitrous oxide as the cryogen which is quite expensive; CryoPop® on the other hand is designed to efficiently use carbon dioxide as the cryogen, and acts by transferring heat from the cervix to the dry ice within the CryoPop® applicator in order to freeze the cervical lesion.^96^, ^104^ This device does not require a CO2 or N2O tank to function, is approximately half the cost of traditional cryotherapy devices, and is more CO2‐efficient by using a tenth of the CO2 per procedure than that of traditional cryosurgery equipment.^99^, ^104^

Breast cancer is the second leading cause of cancer deaths globally, and it accounts for 30% of new cancer diagnosis in women annually. Additionally, more than 40% of women in the United States have dense breast tissue which is reflected by the large quantity of fibrous and glandular tissue rather than fatty tissue. This increased density puts these women at a greater risk of developing breast cancer, and also decreases the visibility of the cancer on traditional mammograms. Especially with the incidence rate increasing by 0.5% per year, it has been a top priority to develop new technologies that can accurately screen these patients. LumaGEM® Molecular Breast Imaging system is an example of one that works by analyzing photons to measure overall radionuclide distribution which aids in the evaluation of breast lesions. It also illustrates areas of enhanced metabolic activity in the breast which is typically not seen on a traditional mammogram due to variable factors such as tissue density and tumor size. Advantages of this system include being cost‐effective, while also being well‐tolerated by patients. Furthermore, the development of this system has addressed the ongoing challenge of detecting small lesions in women who have dense breast tissue; when used as a screening modality in addition to traditional mammography, LumaGEM® has increased the detection rate of early‐stage invasive breast cancers by almost 400%. Another technological advancement is called MarginProbe® which is used during early‐stage breast lumpectomy procedures, and functions to explore the tissue surrounding the malignant cells. The mechanism behind this system centers around the electromagnetic properties of the tissue. It captures and analyzes microscopic differences in electromagnetic properties which is then compared to an internal database of known properties within healthy and cancerous tissues. A major advantage of this system is that it provides real‐time information indicating whether or not the cancer remains at the margins of the resected tissue. This is beneficial for the surgeon in determining if it is necessary to resect additional tissue or to complete the lumpectomy procedure at that point. Lumpectomies have been the gold standard of surgical breast cancer treatment because they have the ability to remove the tumor, while preserving an adequate amount of surrounding healthy tissue. One of the largest challenges, however, is obtaining clean margins which greatly influences the procedure's efficacy. Having this clean margin around the cancerous tissue is crucial to increase the chances of achieving a successful outcome, preventing recurrence, and decreasing re‐excision rates. Dr. Freya Schnabel, the director of breast surgery at NYU Langone's Perlmutter Cancer Center, performed a randomized control trial which concluded that the MarginProbe® decreased the total number of patients requiring second surgeries by 26%. Additionally, the re‐excision rates in her department have been trending down from 24% to 11%; because there is a minimal need for a second surgery to be performed, the overall cost for the patient decreases as well. Incorporation of machine learning algorithms to classify gene expression in low‐cost transcriptional diagnostics has accelerated the diagnosis of lymphomas in LMICs.^105^

Thus, there is a need for more cost‐effective and efficient devices to be implemented within the clinical setting so that physicians can screen for and treat various cancers in a timely manner.

In order to efficiently, accurately, and affordably diagnose diseases, it is essential that we thoroughly understand the current challenges regarding diagnostic tools and start placing more emphasis on the importance of using low‐cost diagnostic and treatment technologies. The development of more low‐cost diagnostics and treatment interventions are needed in order to ensure that a majority of patients have access to these technologies; this may potentially aid in increasing the number of accurate diagnosis made and decreasing the number of precancerous lesions transforming into invasive cancers. Diagnostics has a major role in the forward progression of global health, and targeting this issue has the potential to significantly transform the healthcare setting.

### 4.2 Improvements needed in cancer diagnostics

Cancer diagnostic techniques are constantly undergoing developments and changes in order to successfully fulfill the primary goal of this field of medical care which is to provide accurate diagnosis in a timely manner. However, the presence of ongoing challenges within this field allows for the existence of opportunities to improve certain aspects of cancer diagnostics to quickly identify tumors, and accurately monitor tumor growth and metastasis. The first aspect of cancer diagnostics that can be improved is through the multi‐disciplinary collaboration among radiologists, pathologists, and other involved specialties. Most often, the initial step in the cancer diagnostic process is imaging followed by a tissue biopsy; due to this two‐ step process, constant communication between radiologists and pathologists is necessary to ensure that the results of the biopsy performed directly correlate with the images gathered.^106^ Having multi‐ disciplinary team discussions are crucial for making a definitive diagnosis and rational treatment plan via the combined knowledge and perspectives of the group, and for decreasing the amount of time associated with performing these diagnostic practices.^38^

Another area of cancer diagnostics that could be refined to be more optimally efficient are the systems integrated within the various imaging modalities such as the picture archiving and communication system (PACS).^107^ PACS is a computer network system used for the electronic storage and display of radiologic images rather than manually storing X‐ray films. It is beneficial because it provides convenient storage and access to images from several imaging modalities, replaces conventional films with digital images, and allows the viewing of multiple images at the same time which is not something that can be done with conventional films.^107^ After the imaging is completed, each image must be analyzed in multiple planes and this tends to be a very time‐ consuming process; thus, the more improvements we continue to make in the PACS system and in its interaction with other programs, the more we can facilitate the diagnostic process.^107^ Because PACS is such a useful system, if we can continue to make slight modifications to make it more efficient but economical, we can potentially store radiology reports in a digitally‐organized manner for easy access, better visualize and interpret images since they can be maneuvered through rotating and enlarging, and decrease cost by preventing the need to print films. New ideas, innovations, and enhancements should always be considered when trying to refine current technologies in cancer diagnostic techniques like the PACS system in order to achieve more reliable radiology tools and workflows.

Moreover, due to the ability to deliver multiple ligands and target receptors and other biological factors nanotechnology has proven to be an attractive approach in cancer diagnostics and therapeutics. Various types of nano‐formulations like liposomes, iron oxide, dendrimers, quantum dots, gold nanoparticles, and carbon nanotubes are utilized for diagnostic application in optical, MRI, PET, CT, SPECT, and X‐Rays techniques.^108^ These nanoparticles can be targeted actively or passively targeted into the tumor for imaging and can be used as contrast agents (MRI and photoacoustic tomography). ^111^In‐DTPA‐labeled pegylated liposomes have been used to image different types of cancers (breast brain, head and neck and lung cancer) using SPECT imaging.^108^ ^18^F‐liposomes is used in PET imaging and gadolinium‐loaded nanoparticles in MRI imaging. Nanotechnology is advancing very rapidly and might prove critical in tumor imaging and therapeutics. Through extensive interdisciplinary exchange of information between clinical and basic research will be vital in developing a potential futuristic roadmap, which will extend the realm of early cancer detection and diagnostics via developing technologies targeting vital cellular processes such as hypoxia, angiogenesis, and apoptosis, implicating in the management of refractory tumors.^109^, ^110^, ^111^

## 5 CONCLUSION

It is now well recognized that a proactive approach in the early diagnosis of cancer is key to enhance the efficacy of cancer management by improving the efficacy of most therapies with minimum side effects thereby providing extended survival with good quality of life. Many conceptual and technological advancement that has taken place in the posthuman genomic area coupled with a blast in the computational technologies that the world has witnessed in the last few decades has not only strengthened the classical diagnostic methods that existed for more than several decades, but has also given rise to the development of a number of novel approaches for early, reliable and faster diagnosis of cancer. These include (but not limited to) Fluid or liquid biopsy coupled with novel methods of biomarkers identification comprising cell free DNA and circulating tumor DNA analysis, Exosomes analysis, Molecular cancer diagnostics using DNA microarray, PCR, and Nextgen sequencing as well as long noncoding RNAs (lncRNAs), Molecular cytogenetic techniques like FISH, SKY and so forth. Multi‐parameter digital flow cytometry coupled with multi‐antigen and multi‐spectral probes; risk assessment tools like Mammaprint and Oncotype DX; Applications of nanotechnology and Synthetic biomarkers, Multi‐imaging platforms. More importantly, artificial intelligence (AI) based analysis and integration of complex and large dataset on biomarkers generated by multi‐omic investigations, structural and functional imaging modalities with clinical, pathological and biochemical parameters will play a major role in diagnostics that will have a bearing on the design of novel targeted therapies. Further incorporations of deep learning and convolutional neural networks (CNNs) in variety of different radiological diagnostic modalities extends the realm of tumor imaging, improving image quality and analysis, enhancing early diagnosis, detection and therapeutic outcome.

Taken together the major practices utilized for the diagnosis of cancer, as well as their advantages, limitations, and areas for improvement. While some of these methods have been in use for a long time, new advancements in technology have their own clinical niche. As the field of medicine expands and we continue to strengthen our understanding of cancer, these diagnostic procedures will become more powerful and concomitantly, new techniques will be developed.

## AUTHOR CONTRIBUTIONS

All authors had full access to the data in the study and take responsibility for the integrity of the data and the accuracy of the data analysis. Conceptualization: Rao V. L. Papineni, Akhil Pulumati, Amit Verma, and Bilikere S Dwarakanath; Methodology: Rao V. L. Papineni, Akhil Pulumati, Anika Pulumati, Amit Verma, and Bilikere S Dwarakanath; Investigation: Rao V. L. Papineni, Akhil Pulumati, Amit Verma; Formal Analysis: Rao V. L. Papineni, Akhil Pulumati, Anika Pulumati, Amit Verma, and Bilikere S Dwarakanath; Resources: Rao V. L. Papineni, Akhil Pulumati, Anika Pulumati, Amit Verma; Writing ‐ Original Draft: Akhil Pulumati; Writing ‐ Review & Editing: Rao V. L. Papineni, Akhil Pulumati Anika Pulumati, Amit Verma, and Bilikere S Dwarakanath; Visualization: Rao V. L. Papineni, Anika Pulumati, Amit Verma, and Bilikere S Dwarakanath; Supervision: Rao V. L. Papineni, Amit Verma.

## CONFLICT OF INTEREST

The authors have stated explicitly that there are no conflicts of interest in connection with this article.

## Data Availability

Data sharing is not applicable to this article as no new data were created or analyzed in this study.
